# Intranasal esketamine combined with oral midazolam provides adequate sedation for outpatient pediatric dental procedures: a prospective cohort study

**DOI:** 10.1097/JS9.0000000000000340

**Published:** 2023-06-05

**Authors:** Jing Wang, Jie Zeng, Nan Zhao, Silu Chen, Zhong Chen, Jingrong Liao, Haosong Ran, Cong Yu

**Affiliations:** a Department of Anesthesiology, the Stomatology Hospital Affiliated Chongqing Medical University; b Chongqing Key Laboratory of Oral Diseases and Biomedical Sciences; c Chongqing Municipal Key Laboratory of Oral Biomedical Engineering of Higher Education; d College of Artificial Intelligent, Chongqing University of Technology, No. 69 Hongguang Rd, Banan, Chongqing; e Engineering Research Center of Fujian University for Stomatological Biomaterials, Xiamen Medical College, Xiamen, China

**Keywords:** dental surgery, ED_95_, intranasal esketamine, pediatric, sedation

## Abstract

Severe dental phobia or failure to cooperate with treatment are very common in outpatient pediatric dentistry. Personalized and appropriate noninvasive anesthesia methods can save medical expenses, improve treatment efficiency, reduce the anxiety of children, and improve the satisfaction of nursing staff. Currently, there is little conclusive evidence for noninvasive moderate sedation strategies in pediatric dental surgery.

**Materials and methods:**

The trial was conducted from May 2022 to September 2022. Each child was first given midazolam oral solution 0.5 mg·kg^−1^, and when the Modified Observer’s Assessment of Alertness and Sedation score reached 4, a biased coin design up-down method was used to adjust the dose of esketamine. The primary outcome was the ED_95_ and 95% CI of intranasal esketamine hydrochloride with midazolam 0.5 mg·kg^−1^. Secondary outcomes included the onset time of sedation, treatment and awakening times, and the incidence of adverse events.

**Results:**

A total of 60 children were enrolled; 53 children were successfully sedated but 7 were not. The ED_95_ of intranasal esketamine with 0.5 mg·kg^−1^ midazolam oral liquid for the treatment of dental caries was 1.99 mg·kg^−1^ (95% CI 1.95–2.01 mg·kg^−1^). The mean onset time of sedation for all patients was 43.7±6.9 min. 15.0 (10–24.0) min for examination and 89.4±19.5 min for awakening. The incidence of intraoperative nausea and vomiting was 8.3%. Adverse reactions such as transient hypertension and tachycardia occurred during the operations.

**Conclusion:**

The ED_95_ of intranasal esketamine with 0.5 mg·kg^−1^ midazolam oral liquid for the outpatient pediatric dentistry procedure under moderate sedation was 1.99 mg·kg^−1^. For children aged 2–6 years with dental anxiety who require dental surgery, anesthesiologists may consider using midazolam oral solution combined with esketamine nasal drops for noninvasive sedation after a preoperative anxiety scale evaluation.

## Introduction

Highlights

- Dental anxiety in children is very common and detrimental to surgery.
- Esketamine combined with midazolam is an effective tool for sedation in children.
- The incidence of adverse events of esketamine combined with midazolam for sedation is low.

Dental caries is a multifactorial condition involving the colonization of the oral cavity with cariogenic microorganisms, the use of fluoride, diet, oral health, and the interaction of genetic, biochemical, and social factors^1^. Severe early childhood caries is a major healthcare issue that affects infants and preschool children globally, with its prevalence in China continuing to increase^2–4^. If treatment is delayed, it will cause pain and infection, and even cause alveolar and jaw bone inflammation, leading to tooth loss^5^, eventually causing eating difficulties, language and cosmetic changes in children’s later growth and development. In most cases, a pediatric dental procedure is performed under local anesthesia in a dental surgery setting. Nondrug behavior management, especially tell-show-do are regular methods used to improve the degree of cooperation during dental procedures. However, severely anxious children or special-needs children (more common in preschool children) are fearful and lack cooperation during the dental procedure. Failure to cooperate with dentists and a lack of sufficient treatment time can seriously limit the effects of the dental treatment. Treatment of Severe early childhood caries under general anesthesia is considered to be the best drug behavior management approach for young children and is increasingly recommended by dentists in China, but there are associated medical risks and increased costs. Therefore, we explored an easy approach to implement a moderate sedation scheme suitable for short dental treatment^6,7^. At the present time, sedatives administered to children (such as propofol, etomidate, pentobarbital) require invasive procedures involving intravenous access. About one percent of infants and children are given dexmedetomidine^8^ with the possibility of eliciting life-threatening adverse events (AEs). About 5% of patients given intravenous short-acting barbiturates and circa 7% intravenous midazolam experienced AEs, notably respiratory depression, and transient desaturation^9,10^. Effective local or regional anesthesia usually reduces the dose of sedative required and improves the safety of the surgical procedure^11^. Although midazolam oral solution and dexmedetomidine have good sedative effects, they do not have analgesic effects. Esketamine is the dextrorotatory structure of racemic ketamine, and blocks N-methyl-D-aspartic acid activity in the central nerve system, inducing sedation, analgesia, and unconsciousness. Its affinity for the N-methyl-D-aspartic acid receptor is higher, and is 2 times more potent in its anesthesia and analgesia effects compared to ketamine. In addition, the mental symptoms are milder, and it is especially suitable for inducing short-term surgical anesthesia in children without the need for endotracheal intubation^12^. Nasal administration is an excellent route for children, is simple and has a high bioavailability^13,14^. However, children treated with esketamine alone have exhibit increases in heart rate and blood pressure, and the rapid heart rate can cause agitation and endanger the child’s life. Therefore, esketamine should not be used alone in pediatric anesthesia^15^. As a new generation product, the actions of esketamine need to be further investigated. The ED_95_ can reflect the effect of the continuous action of the drug, which is not normally investigated in controlled studies. Our aim was to provide the interested reader with the most effective dose in which the desirable clinical effect elicited will be certain. Midazolam oral liquid is a short-acting benzodiazepine with sedative, amnesic, and anxiolytic effects, usually prescribed as preoperative medication for children^16^. In terms of drug interactions, the net effect of intranasal esketamine on midazolam is short-term weak inhibition, with an increase of less than 1.5 times and no clinical significance^17^. Therefore, we use midazolam oral solution combined with esketamine nasal drops^18^. This methodologic improvement study was designed to determine the ED_95_ of intranasal esketamine with 0.5 mg·kg^−1^ midazolam oral liquid as a moderate sedation regimen for the treatment of children’s dental caries by the biased coin design up-down method (BCD-UMD).

## Materials and methods

### Study design

The trial was conducted from May 2022 to September 2022. The children underwent pediatric dental surgery including caries treatment and complex supernumerary tooth extraction under moderate sedation induced with midazolam and intranasal esketamine. The study was carried out in strict accordance with the Declaration of Helsinki principles for research on humans and was approved by the Institutional Review Board of Stomatological Hospital Affiliated to Chongqing Medical University, Chongqing, China April 23, 2022 [No.: CQHS-REC-2022(LSNo.132)]. All children’s legal representatives signed informed consent. The trial was registered at chictr.org.cn before the patients were enrolled (ChiCTR2200058047, registration date: 27 March 2022). This study has been reported in accordance with strengthening the reporting of cohort, cross-sectional and case-control studies in surgery (STROCSS) standards^19^. Supplemental Digital Content 1, http://links.lww.com/JS9/A649.

### Study population

The trial inclusion criteria were: children aged 2 to 6 years old who were scheduled to receive sedation for dental surgery; American Society of Anesthesiologists medical status I to II. The exclusion criteria were: nonconsensual; known to be allergic to midazolam oral solution and/or esketamine; hepatic or renal problems; runny nose; serious systemic disease (for example: congenital heart disease; Kawasaki disease; multisystem inflammatory syndrome; primary systemic vasculitis); or a diagnosis of fever.

### Study procedures and interventions

All patients fasted for greater than or equal to 2 h. Oral midazolam solution and the esketamine intranasal instillation were administered by an anesthesiologist who was not involved in collecting trial data. A dose of 0.5 mg·kg^−1^ midazolam oral solution and nasal drops of undiluted esketamine (2 mg·kg^−1^) were employed.

**Figure 1:**
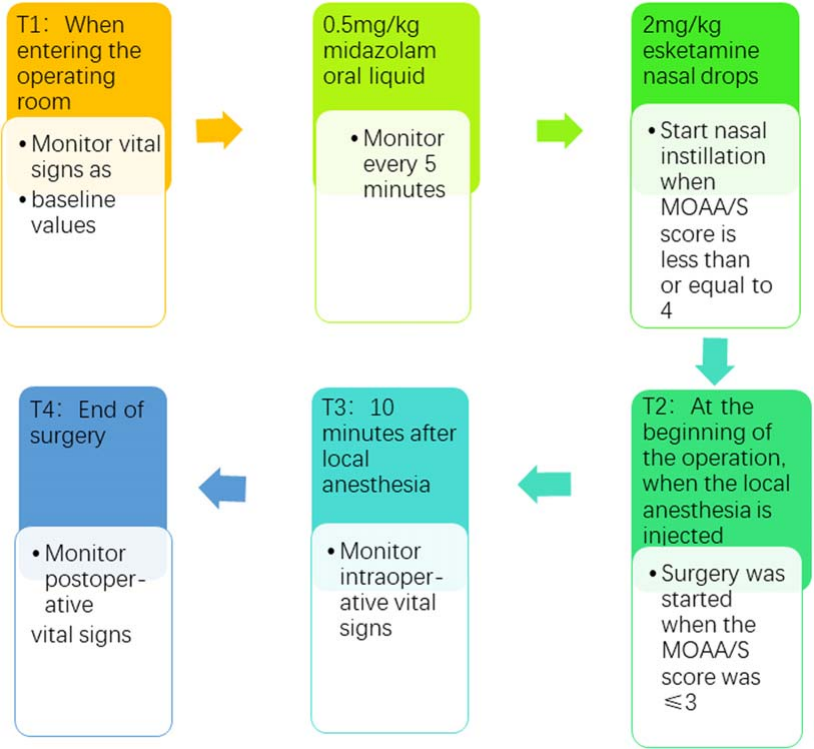
Operation time chart.

Children who visited the clinic on the same day were evaluated by anesthesiologists, and informed consent was obtained from the children’s guardians. Before patients entered the treatment room, the following vital signs were measured: heart rate; blood pressure; and blood oxygen saturation. Sedation was assessed by a blinded anesthesiologist using the Modified Observer’s Assessment of Alertness and Sedation (MOAA/S)^20,21^ (eAppendix 1, Supplemental Digital Content 2, http://links.lww.com/JS9/A650) scale every 5 min after the administration of midazolam oral solution. After administration, our anesthesiologist stood on the right side of the patient, called out the patient’s name, and judged the depth of sedation based on the patient’s response to their name. When we called a patient’s name in a normal voice, they were unresponsive, and the MOAA/S score was 4 at this time. Until we needed to call out their names loudly and/or repeatedly. Only then could patients respond, at which point the MOAA/S score was 3. Esketamine intranasal instillation was performed when the MOAA/S sedation score reached 4. Immediately after successful sedation, the pediatric dentist infiltrated the surgical area with local anesthetic. If the patient was less than 4 years old they were given an injection of 2% lidocaine (5 ml: 0.1 g, Southwest Pharmaceuticals; SFDA No. H50020038); the maximum total dosage was <4 mg·kg^−1^. Patients greater than or equal to 4 years old were given 4% articaine and epinephrine injection (1.7 ml: 68 mg, Products Dentaires Pierre Rolland; SFDA No. H20140732) up to a maximum dosage of 5 mg·kg^−1^. Five minutes after local anesthetic injection, the treatment procedure was initiated. To prevent aspiration of water ingress and foreign objects, rubber dams were placed for isolation during the surgical procedure. The onset time of sedation was defined as the time when patients had MOAA/S scores less than or equal to 3. The success of sedation was defined as: under the condition of MOAA/S less than or equal to 3, the operation was successfully completed without remedial measures. And the marker of sedation failure were MOAA/S score greater than or equal to 4 points. If the patient failed to be sedated, a salvage ‘sevoflurane inhalation’ remedy were given to complete the dental surgery. Total sevoflurane inhalation induced sedation deep has been widely used in our dental procedures, and our previous clinical trial revealed that solo sevoflurane exposure did not lead to adverse neurocognitive outcomes or neurological deficits in healthy preschool children (Fig. 1)^22^.

According to previous relevant literature and our preliminary trials^23^, we choose 0.5 mg·kg^−1^ as constant dose of midazolam oral solution (10 ml: 20 mg, Yichang Humanwell, Inc., YiChang, HuBei; SFDA No. H20213321), the initial dose of intranasal esketamine is 1 mg·kg^−1^ as followed (2 ml: 50 mg, Jiangsu Hengrui Medicine; SFDA No. H20193336). The next dose was based upon previous patient responses, using the BCD-UMD^24^. The dosing interval was 0.25 mg·kg^−1^. If sedation failed, the next patient’s dose of esketamine was increased by 0.25 mg·kg^−1^, and if sedation was successful, the next patient was randomized to receive the same dose (*P*=0.95), or 0.25 mg·kg^−1^ less (*P*=0.05). It is fair to say that the sample size for the BCD-UMD has not been well defined, but anesthesia trials that employ the top-down approach typically have a cohort of 20–40 patients^24^. We recruited 60 patients, including 42 for caries treatment and 18 for supernumerary tooth extraction^25^. The time to onset of sedation was defined as the time from the initiation of the administration of midazolam oral solution to the successful induction of sedation. The wake-up time was defined as the time from intranasal esketamine titration until patient discharge from the dental office. An aldrete score of 9 or above will allow discharge (eAppendix 2, Supplemental Digital Content 3, http://links.lww.com/JS9/A651)^26^. After the dental surgery was finished, the patient was transferred to a recovery area. Patients were followed up with by an original developed smartphone-based applet WeChat Applet for dental anxiety the next day to record any AEs and intelligent feedback (eFigure 1, Supplemental Digital Content 4, http://links.lww.com/JS9/A652)^27^.

### Outcome measure

The primary outcome was the dose from the start of administration to the time when the patient fell asleep (MOAA/S ≤ 3 points). Secondary outcomes included the time to onset of sedation, time to treatment, time to awakening, security indicators (e.g., incidence of AEs such as hypotension, hypertension, bradycardia, tachycardia, respiratory depression, oxyhemoglobin desaturation, reflux and vomiting, hypoglycemia), and sex-specific onset and recovery times.

### Statistical analysis

Pace *et al.* ^24^ recommended the enrollment of 20–40 patients in BCD-UMD based sequential dose-finding studies, as most practical cases provide a stable estimate of the dose; in our study, 60 patients were included. ED_95_ in pediatric patients was estimated using an isotonic regression estimator μ3, and ~95% CIs for ED_95_ were calculated using the bias-corrected percentile method derived from bootstrapping, with a resampling size of 60, the number of replicates 2000 and a target Γ of 0.95^24,28,29^. Adjusted response probabilities were obtained by the Aggregate Neighbor Violator Algorithm. Statistical analysis was performed using Python 3.10 (numpy package: the fundamental package for scientific computing; scipy package: Compute a two-sided bootstrap CIs of a statistic; pandas package: data form management).

Data with a normal distribution are given as the mean±SD, and non-normally distributed data as [medians (interquartile range)], evaluated using SPSS for Windows (version 22.0).

## Results

### Patients’ characteristics and operative data

**Figure 2:**
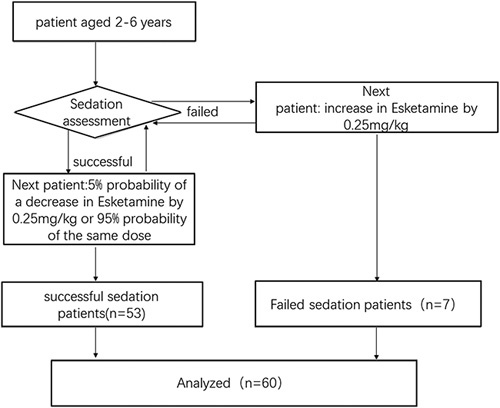
CONSORT flow diagram.

Sixty patients participated in our prospective study, with 53 successful and 7 unsuccessful sedations (Fig. 2). Table 1 provides an overview of the detailed participant demographics, which were similar across groups.

**Table 1:** Characteristics of participants.

Characteristic	Total (*n*=60)	Success (*n*=53)	Failure (*n*=7)
Male, No. (%)	27 (45)	24 (45.3)	3 (42.9)
Female, No. (%)	33 (55)	29 (54.7)	4 (57.1)
Age, year, median (IQR)	51.5 (34.5–64.0)	50 (32.0–63.5)	57 (38–74)
BMI, median (IQR)	17 (16–18)	17.17 (16.3–18.0)	17.35 (16.3–17.7)

### Primary outcome

The ED_95_ of intranasal esketamine after 0.5 mg·kg^−1^ oral midazolam for pediatric dentistry procedure under moderate sedation was 1.99 mg·kg^−1^ with 95% CI of 1.95–2.01 mg·kg^−1^.

The frequency of treatment and observed pool adjacent violator algorithm adjusted response rates for each specific esketamine dose are shown in Table 2. Pool adjacent violator algorithm adjusted response rates were never observed to decrease with increasing dose.

**Table 2:** Observed and PAVA adjusted esketamine response rates (isotonic regression).

Assigned dose (μg/kg)	Number sedative	Number tested	Observed response rate	PAVA adjusted response rate
1	0	1	0	0
1.25	0	1	0	0
1.5	6	8	0.75	0.75
1.75	20	22	0.909	0.909
2	20	21	0.9523	0.9523
2.25	7	7	1	1

### Secondary outcomes

Table 3 details the sedation onset time of 43.7±6.9 min for all patients. Onset time of sedation: 44.1±5.8 min for successful sedation, 15.0 (10–24.0) min for examination, 89.4±19.5 min for awakening.

**Table 3:** Secondary outcome statistics.

Characteristic	Total (*n*=60)	Success (*n*=53)	Failure (*n*=7)
Examination time, median (IQR), min	/	15.0 (10–24.0)	/
Midazolam onset time, median (IQR), min	17.6 (15–20)	17.6 (15–20)	19.6 (16–24.6)
Sedation onset time, Mean±SD, min	43.7±6.9	44.1±5.8	39.3±5.3
Time of wakefulness, Mean±SD, min	/	89.4±19.5	/

### Security indicators

Table 4 details the security indicators. The incidence of nausea and vomiting in all children was 8.3%, the sedation success rate was 7.5%, and the sedation failure rate was 14.3%. The incidence of hypoglycemia in all children was 1.6%, the sedation success rate 1.9%, the incidence of intraoperative hypertension in all children 5%, and the sedation success rate 5.7%. The incidence of tachycardia in all children was 8.5%, the sedation success rate was 1.9%, and the sedation failure rate was 57.1%. There were no AEs such as hypotension, bradycardia, respiratory depression, or oxyhemoglobin desaturation. eFigure 2, Supplemental Digital Content 5, http://links.lww.com/JS9/A663 shows the patient’s heart rate, blood pressure, and oxygen saturation at different times.

**Table 4:** Security indicators.

Characteristic	Total (*n*=60)	Success (*n*=53)	Failure (*n*=7)
Nausea, vomiting	5 (8.3%)	4 (7.5%)	1 (14.3%)
Hypoglycemic reaction	1 (16.7%)	1 (1.9%)	0 (0)
Intraoperative hypertension	3 (5%)	3 (5.7%)	0
Intraoperative tachycardia	5 (8.3%)	1 (1.9%)	4 (57.1%)
Oxyhemoglobin desaturation	0	0	0
Hypotension	0	0	0
Bradycardia	0	0	0
Houpt scale score	5 (5–6)	5 (5–6)	3 (1–3)
MOAA/S scale scores	3 (3–3.8)	3 (3–4)	4 (4–5)

eAppendix 3, Supplemental Digital Content 6, http://links.lww.com/JS9/A664 shows the onset times of sedation for 53 children who were successfully sedated: 44.0±5.4 min for males and 44.5±6.2 min for females, with a *P*-value of 0.762. Wake-up time was 84.9±16.8 for males and 92.8± 16.8 for females, with a *P*-value of 0.146.

**Figure 3:**
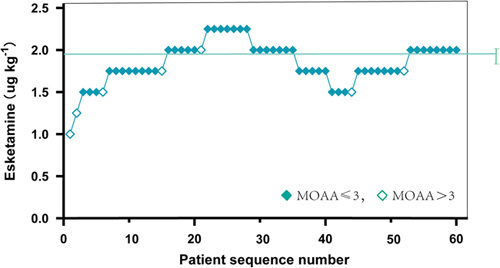
Determination of the ED_95_ of intranasal esketamine in infants receiving dental surgery. Patient serial number (*x*-axis) and the dosage of esketamine dispensed (*y*-axis) are shown. Sedation failures (open squares), successful sedations (solid squares).

The intranasal esketamine doses in the seven patients with sedation failure were 1 mg·kg^−1^, 1.25 mg·kg^−1^, 1.5 mg·kg^−1^, 1.75 mg·kg^−1^, 2.0 mg·kg^−1^ and 1.5 mg·kg^−1^, 1.75 mg·kg^−1^ (Fig. 3). Seven patients with sedation failure, all had MOAA/S score less than or equal to 3 within 30 min of esketamine administration and reached the level of sedation, two patients woke up during local anesthesia and refused treatment (MOAA/S >4), five patients on intraoperative awakening (MOAA/S >3) were administered sevoflurane for inhalation rescue.

## Discussion

Outpatient pediatric dental procedure under anesthesia and sedation, whether in China or other countries, severe hypoxia occurred every few years, mostly due to respiratory-related AEs such as laryngospasm, airway obstruction, and respiratory suppression^30^. Therefore, research on the ratio of anesthetics other than general anesthesia and deep sedation is imminent. Several previous studies have found that esketamine 1 mg·kg^−1^ intranasally is approximately as effective as ketamine 2 mg·kg^−1^ intranasally^31^, whereas ketamine 3 and 6 mg·kg^−1^ did not produce sufficient sedation^32^. Preoperative nasal sedation with 3 mg·kg^−1^ esketamine alone was insufficient for sedation and resulted in a higher incidence of postoperative agitation. Intranasal esketamine can provide adequate sedation for pediatric patients in outpatient care. Higher percentile effective doses provide more clinical guidance, so we explored the ED_95_ of esketamine combined with midazolam oral solution. An isotonic regression estimator with exmedetom statistics that measures responses at arbitrary points (quantiles) on the dose-response curve with low bias and variance. The BCD design approach avoided unvalidated extrapolation of the ED_50_ because most of the peak distributions of administered doses were around the mean. Therefore, we used the BCD design method and the isotonic regression estimator to study the ED_95_ of esketamine combined with midazolam oral solution. In our study, we calculated the ED_95_ of esketamine nasal drops combined with 0.5 mg·kg^−1^ midazolam oral solution to be 1.99 mg·kg^−1^ [95% CI, 1.95–2.01 mg·kg^−1^], the results may provide clinical guidance for performing these pediatric surgeries in children 2–6 years of age undergoing dental surgery.

The combination of esketamine and sedative drugs can be expanded or substituted for other pediatric procedures requiring MOAA/S less than or equal to 3. On the one hand, it is used for pre-examination sedation, such as improving the cooperation of children with inhalation anesthesia masks^31^ better sedation in children with tonsillectomy^33^. Facilitate pediatric upper gastrointestinal endoscopy^34^ and pediatric MRI examination^35^. On the other hand, it has been applied to sedation for other pediatric outpatient procedures, such as: hernia in children, circumcision, debridement, and suture.

It is an important clinical context to provide safe and effective preoperative medication to children, promote their calmness and cooperation, and avoid anxiety and agitation. Although many drugs and methods have been studied worldwide, various details should be highlighted. For example, midazolam oral solution alone is prone to elicit complications such as excitement, hallucinations, behavior changes, and delayed awakening^36^. The sedative effect may even be unsatisfactory due to individual differences in the first-pass effect or AEs such as a high incidence of nausea and vomiting. Increased respiratory secretions may occur when intravenous propofol is combined with different doses of ketamine^37^. Numerous studies have shown that dexmedetomidine is a good alternative for preoperative sedation or sedation before computed tomograhy and other medical imaging examinations, but it does not have an analgesic action. Dexmedetomidine should be used with caution in patients less than 18 years old^38^.

Based on clinical experience and related studies, we gave a dose of midazolam oral liquid of 0.5 mg·kg^−1^. Esketamine is extensively metabolized by cytochrome P450 enzymes, mainly 3A4 and 2B6^39^. N-demethylation to norketamine is the main pathway for the metabolism of esketamine^40^. Norketamine is further metabolized via a cytochrome P450 enzymes-dependent pathway, with an increase in maximum concentration from zero to infinity (C_max_) and the AUC higher than for esketamine, especially after the oral route of administration, so esketamine effects lasted longer ketamine^41,42^. Carlos Perez-Ruixo^43^ stated that the absolute bioavailability of 56 mg and 84 mg intranasal esketamine was 54 and 51%, respectively. Yoshitsugu Yanagihara^44^ estimated that the bioavailability of ketamine administered by nasal spray to be about 45%, so we specified the initial nasal dose as 1.0 mg·kg^−1^ based on the above studies.

In children requiring fractionated diagnosis and treatment, esketamine did not accumulate in the plasma following repeated or infrequent administration after twice weekly intranasal doses^45^. Following intranasal administration, the expected time to reach peak plasma concentrations of esketamine was 20–40 min after the last nasal spray treatment, followed by a rapid decline in the esketamine plasma concentration, with a terminal half-life between 7 and 12 h^46^. The onset time of the drug in this study was 44.2±5.8 min. Considering the onset time of midazolam oral liquid, it was basically consistent with this conclusion.

Combination therapy not only improved patient satisfaction with preanesthetic sedation, but did not increase the wake-up time or the incidence of AEs^47^.

In this trial, the incidence of intraoperative hypertension was 5% and the incidence of tachycardia was 8.3%. The next day, by follow-up with an original smartphone-based applet WeChat applet for dental anxiety, postoperative blood pressure, and heart rate had returned to normal, which were consistent with Doherty’s^15^ findings that blood pressure increases after esketamine administration are usually transient in nature, asymptomatic, and did not associate with serious cardiovascular safety sequelae. In general, AEs related to blood pressure, abnormal heart rate, and cardiovascular. AEs began shortly after dosing in esketamine-treated patients and resolved within 1.5 h of patient dosing^15^. Intraoperative hypertension and tachycardia may also be caused by a 4% articaine injection containing 1:100 000 epinephrine. The incidence of drug induced postoperative nausea and vomiting cannot be unequivocally established because of blood or inflammatory stimuli after oral dental^48^, which differ from other operations. One successfully sedated patient developed hypoglycemia 1 h after surgery. The family was informed that it probably developed because she has fasted for more than 12 h. Studies have shown that esketamine has a lower adverse reaction rate, a shorter recovery time, and directional recovery time than racemic ketamine^49^. The results of our study showed that the onset time of sedation for boys and girls were: 43.0±5.8 min and 44.2±6.0, *P*=0.762, but statistical significance was not reached; the awakening time was 84.9±16.8 and 92.8±21.2 min, respectively, *P*=0.146, which was not statistically different, findings consistent with the result of Jing Wang *et al*.^50^ who reported no sex difference in esketamine efficacy.

### Limitations

Our study had several limitations: The heart rate, blood pressure, respiratory rate, and pulse oximeter measured at baseline before dosing may have been inaccurate due to the children’s anxiety and crying; Several factors may have affected the level of sedation, including the duration of fasting before sedation, the child’s sleep quality. It is noteworthy that the seven patients who failed sedation slept well the night before sedation. More data are needed in future studies.

## Conclusions

The ED_95_ of intranasal esketamine with 0.5 mg·kg^−1^ midazolam oral liquid for the outpatient pediatric dentistry procedure under moderate sedation was 1.99 mg·kg^−1^. Such regimens provide safe and moderate sedation for pediatric dental procedure in an office-based setting.

## Ethical approval

This study was approved by the Institutional Review Board Stomatological. Hospital Affiliated to Chongqing Medical University, Chongqing, China 23 April 2022 (No.: CQHS-REC-2022 (LSNo.132)).

## Sources of funding

The authors disclose that they received the following financial support for the research publication of this article. Fujian Oral Biomaterials College (Xiamen Medical College) Engineering Research Center. (Grant No.: XMMC-KQ202102); Intelligent Medicine Project of Chongqing Medical University (Grant No.: ZHYX202116).

## Author contribution

C.Y.: concept and design; J.W., J.Z., N.Z., S.I.C., Z.C., and J.L.: acquisition, analysis, or interpretation of data; J.W.: drafting of the manuscript; C.Y.: critical revision of the manuscript for important intellectual content; J.Z., H.S.R.: statistical analysis.

## Conflicts of interest disclosure

None reported.

## Research registration unique identifying number (UIN)

1. Name of the registry: Chinese Clinical Trial Registry chictr.org.cn.

2. Unique Identifying number or registration ID: ChiCTR2200058047.

3. Hyperlink to your specific registration (must be publicly accessible and will be checked): https://www.chictr.org.cn/showprojen.aspx?proj=155738.

## Guarantor

CongYu, JingWang, JieZeng.

## Data availability statement

Informed consent has been signed for this clinical trial, and the original data will not be shared.

## Provenance and peer review

Not commissioned, externally peer-reviewed.

## Supplementary Material
