# Supplementary material for: Intranasal esketamine combined with oral midazolam provides adequate sedation for outpatient pediatric dental procedures: a prospective cohort study
Source: Int J Surg. 2023 Jun 5;109(7):1893–9. doi: 10.1097/JS9.0000000000000340 (PMC10389564; doi:10.1097/JS9.0000000000000340)
Supplement: Supplementary file 2 [file js9-109-1893-s002.docx]

**eAppendix 1. Modified observer's assessment of alertness/sedation scale.**

|  | Score |
| --- | --- |
| Does not respond to a noxious stimulus | 0 |
| Does not respond to mild prodding or shaking | 1 |
| Responds only after mild prodding or shaking | 2 |
| Responds only after name is called loudly and repeatedly | 3 |
| Lethargic response to name spoken in normal tone | 4 |
| Appears asleep but responds readily to name spoken in normal tone | 5 |
| Appears alert and awake and responds readily to name spoken in normal tone | 6 |

The marker of sedation success were MOAA/S scores ≤ 3 points and the marker of sedation failure were MOAA/S score ≥ 4 points.
