# Supplementary material for: Intranasal esketamine combined with oral midazolam provides adequate sedation for outpatient pediatric dental procedures: a prospective cohort study
Source: Int J Surg. 2023 Jun 5;109(7):1893–9. doi: 10.1097/JS9.0000000000000340 (PMC10389564; doi:10.1097/JS9.0000000000000340)
Supplement: Supplementary file 3 [file js9-109-1893-s003.docx]

**eAppendix 2 Modified Aldrete score**

|  | Score |
| --- | --- |
| State of activity |  |
| Able to move four extremities voluntarily or on command | 2 |
| Able to move two extremities voluntarily or on command | 1 |
| Able to move 0 extremities voluntarily or on command | 0 |
| State of respiration |  |
| Able to breathe deeply and cough freely | 2 |
| Dyspnoea or limited breathing | 1 |
| Apnoeic | 0 |
| State of circulation |  |
| BP ± 20 mmHg of preanaesthetic period | 2 |
| BP ± 20–50 mmHg of preanaesthetic period | 1 |
| BP ± 50 mmHg of preanaesthetic period | 0 |
| State of consciousness |  |
| Fully awake | 2 |
| Arousable on calling | 1 |
| No response | 0 |
| State of O2 saturation |  |
| >92% on room air | 2 |
| **Needs O_2_ to maintain SpO_2_ > 90%** | 1 |
| **<90% despite O_2_ supplementation** | 0 |

SpO_2_, oxygen saturation. Aldrete score of 9 or above will allow discharge.
