# Supplementary figures and images for: Intranasal esketamine combined with oral midazolam provides adequate sedation for outpatient pediatric dental procedures: a prospective cohort study
Source: Int J Surg. 2023 Jun 5;109(7):1893–9. doi: 10.1097/JS9.0000000000000340 (PMC10389564; doi:10.1097/JS9.0000000000000340)

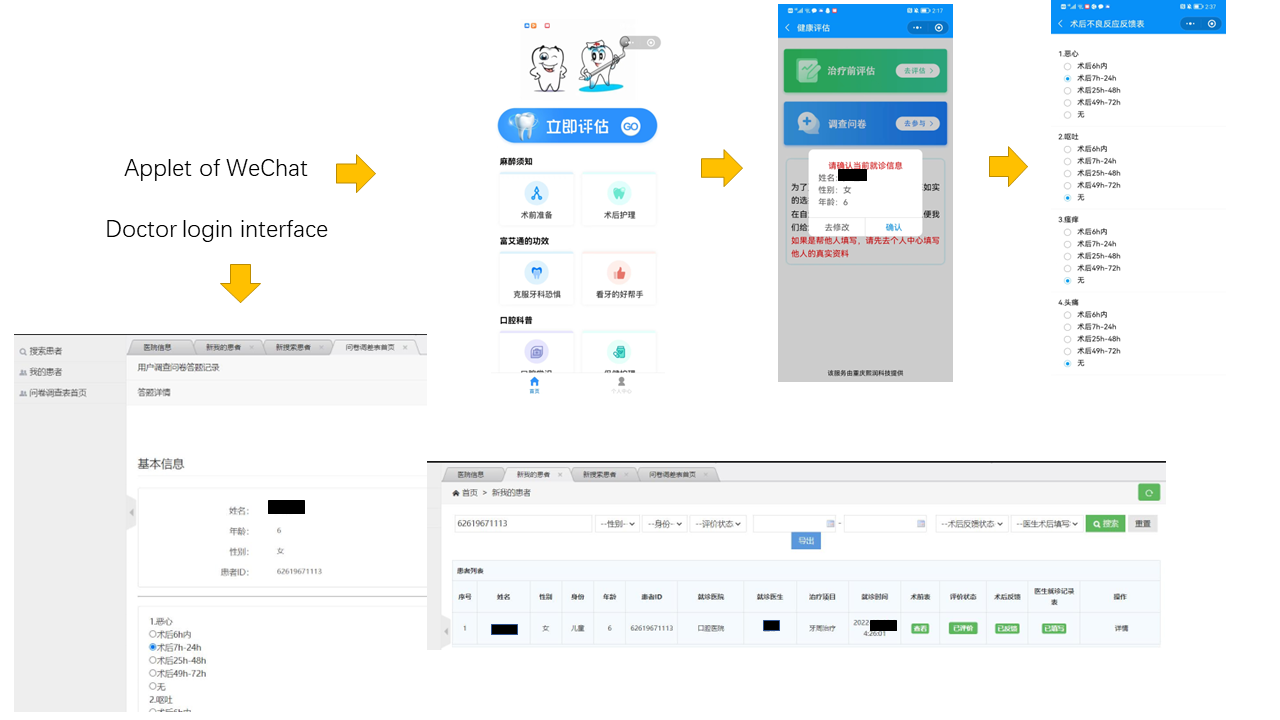

Supplement: Supplementary file 4 [file js9-109-1893-s004.tif]

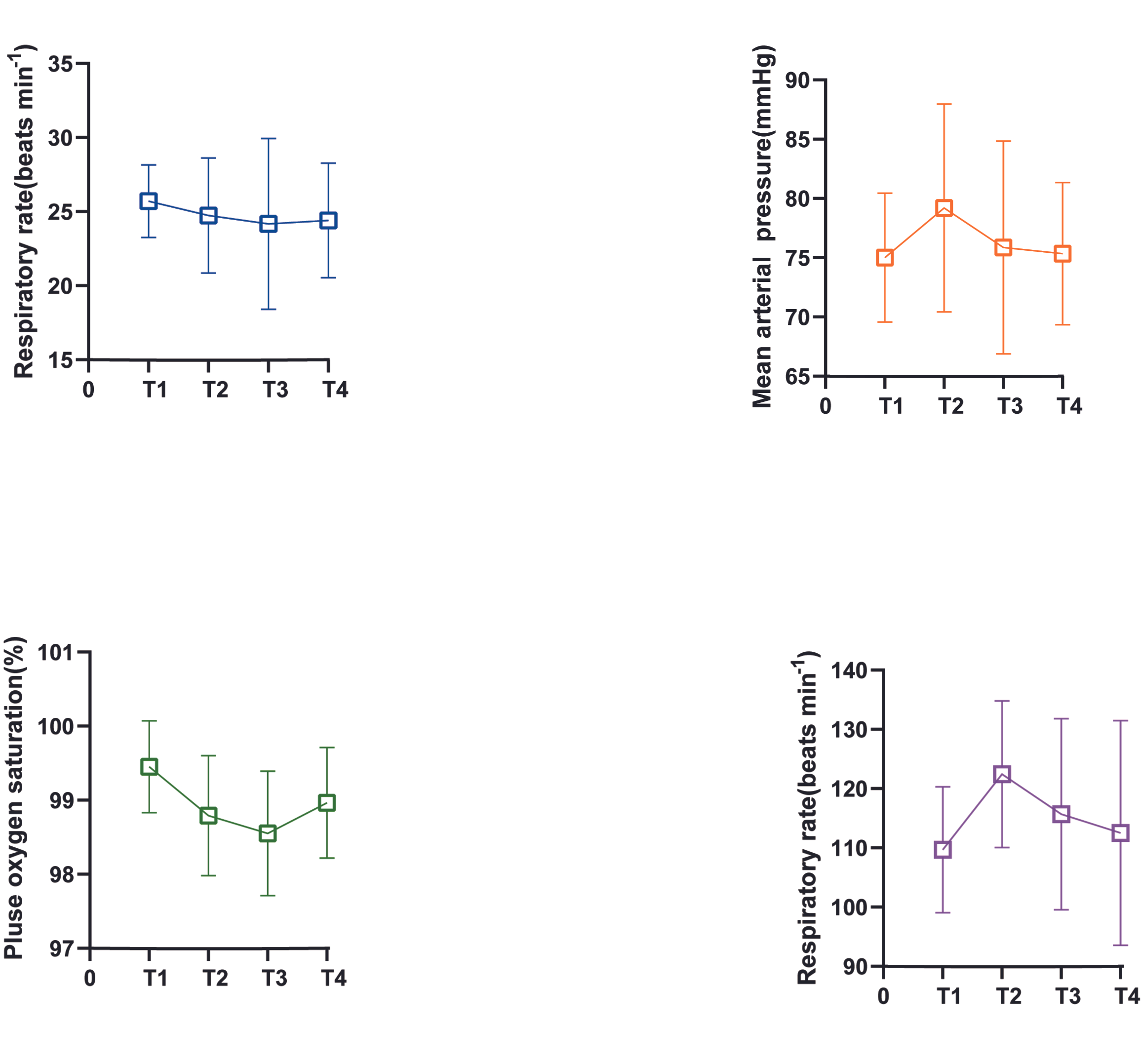

Supplement: Supplementary file 5 [file js9-109-1893-s005.tiff]
