# Supplementary material for: Intranasal esketamine combined with oral midazolam provides adequate sedation for outpatient pediatric dental procedures: a prospective cohort study
Source: Int J Surg. 2023 Jun 5;109(7):1893–9. doi: 10.1097/JS9.0000000000000340 (PMC10389564; doi:10.1097/JS9.0000000000000340)
Supplement: Supplementary file 6 [file js9-109-1893-s006.docx]

**eAppendix 3 shows the different gender comparisons were analyzed using Student's t-test**

| **Sex** | **Male (n = 24)** | **Female (n = 29)** | ***P*-value** |
| --- | --- | --- | --- |
| Sedation onset time | 44.0 ± 5.4 | 44.5 ± 6.2 | 0.762 |
| Arousal time | 84.9 ± 16.8 | 92.8 ± 21.2 | 0.146 |
